# Intergenerational healthcare ethics: considering conceptualizations of generations and their collective and temporal dimensions

**DOI:** 10.1007/s11019-025-10297-0

**Published:** 2025-12-15

**Authors:** Niklas Ellerich-Groppe, Claudia Bozzaro, Dominik Koesling, Christoph Rehmann-Sutter, Silke Schicktanz, Mark Schweda

**Affiliations:** 1 Division of Ethics in Medicine, Department for Health Services Research, Carl von Ossietzky Universität Oldenburg, Oldenburg, Germany; 2 Institute for Ethics, History and Theory of Medicine, University of Münster, Münster, Germany; 3 Institute for History of Medicine and Science Studies (IMGWF), University of Lübeck, Lübeck, Germany; 4 Department for Medical Ethics and History of Medicine, University Medical Center Göttingen, Göttingen, Germany

**Keywords:** Healthcare ethics, Medical ethics, Generation, Collectivity, Temporality, Time, Future, Intergenerational bioethics

## Abstract

Current challenges in medicine and healthcare raise new questions regarding the moral relations between generations, thus highlighting the increasing relevance of intergenerational perspectives in healthcare ethics. However, the underlying notions of generations often remain vague and heterogeneous. This contribution aims to clarify the scope of conceptual meanings of ‘generation’ through explication and differentiation in order to advance the analytical potential of intergenerational perspectives in healthcare ethics. We argue that the concept of generations needs theoretical elaboration with regard to the dimensions of collectivity and temporality. We first introduce three approaches towards the theoretical conceptualization of generations: a genealogical, a chronological, and a socio-cultural approach. Regardless of their differences, all three essentially share an understanding of generations as collectives situated in time. Accordingly, we then examine the scope of underlying notions of collectivity and temporality, touching upon fundamental ontological, epistemological, and moral philosophical implications. We distinguish a skeptical individualist, an aggregationist, and an entity view of collectivity, as well as a formal, linear, a subjective, existential-narrative, and a socio-cultural understanding of temporality. The combination of these dimensions allows the development of a systematic matrix of conceptions of generations and intergenerational relations in healthcare ethics whose analytical potential we illustrate with regard to three paradigmatic examples. We provide a systematic summary of our considerations and outline a research agenda that addresses desiderata for intergenerational perspectives in healthcare ethics, encompassing clinical ethics, research ethics, and public health ethics, as well as meta-ethical questions.

## Introduction

Challenges like population aging and climate change raise new ethical issues regarding intergenerational relations in medicine and healthcare (Macpherson 2014). The ensuing discussions center on the moral relationships between generations and their mutual claims and responsibilities. Such intergenerational issues have become increasingly topical in various fields of healthcare ethics, comprising clinical ethics, medical research ethics, and public health ethics.

One prominent example in *clinical ethics* is the antibiotic crisis. The excessive use of antibiotics leads to the spread of antimicrobial resistance, posing moral conflicts “between the welfare of the present patient and that of future, unidentified persons” (Leibovici et al. 2012, p. 13). In order to address the question “[i]f current patterns of antibiotic use cannot be sustained for future generations” (Millar 2011, p. 156), however, a clear concept of what constitutes a generation is needed.

The field of *research ethics* provides further examples. The collection and long-term use of big data sets, including genomic data, raise ethical issues regarding intergenerational relations. Thus, data protection efforts in research can no longer focus on “the single research subject but [must] extend[] gained knowledge and its consequences to genetically related persons and thus future generations” (Brall et al. 2017, p. 30). Yet, the concrete composition, characteristics, and moral relevance of these future generations require further explication.

The COVID-19 pandemic represents a third example from the field of *public health ethics*. The notion of intergenerational solidarity appeared to be almost ubiquitous in many debates during the pandemic. Thus, politicians as well as researchers emphasized “the importance of intergenerational solidarity and the neglected support of the younger generation” (Schönweitz et al. 2024). However, to clarify the scope and justification of moral responsibilities between generations suggested in such appeals to intergenerational solidarity, the underlying concept of generations and their moral relations need clarification.

These examples underscore the growing significance of intergenerational perspectives in healthcare ethics. They illustrate that certain moral questions and conflicts in clinical practice, medical research, and public health point beyond individual (inter-)actions at a given point in time and therefore call for a more encompassing intergenerational framework. At the same time, the given examples highlight the need for more clarity, consistency, and differentiation in how notions of generations and their interrelations are conceptualized in the accompanying ethical debates.

Our contribution aims to clarify the concept of generations in order to advance the theoretical foundations of intergenerational perspectives in healthcare ethics. We first introduce three approaches to the conceptualization of generations and intergenerationality and underline their potential for healthcare ethics (“Background: what is a generation?” section). We then point to the relevance of collective and temporal dimensions and distinguish underlying understandings of collectivity and temporality that can help to explicate, systematize, and reflect notions of generations and to address relevant questions in intergenerational healthcare ethics in a more precise way (“Generations as collectives situated in time: towards a theoretically enriched understanding” section). On this basis, we present a systematic matrix of conceptions of generations with respect to their collective and temporal dimensions and illustrate its analytical value for debates on intergenerational relations in healthcare ethics with regard to three paradigmatic examples (“Paradigms of intergenerationality: framing moral relations between generations” section). Concluding, we summarize the benefits of a theoretically enhanced understanding of generations and intergenerationality in healthcare ethics and outline a research agenda that addresses desiderata regarding clinical ethics, research ethics, and public health ethics, as well as meta-ethical questions (“Conclusion: what intergenerational perspectives can contribute to healthcare ethics” section).

## Background: what is a generation?

The concept of generations has multiple meanings (Kertzer 1983, p. 125). Based on the pertinent literature from social science and cultural studies, at least three main theoretical strands can be distinguished. Each conceptualizes generations in a different way (for overviews, see Kertzer 1983; Bengtson and Oyama 2010; Höpflinger 2012; Costanza et al. 2023).

In a *first*, *genealogical or familial-kinship interpretation*, the concept of generations refers to a sequence of individuals or collectives connected by descent or succession (Höpflinger 2012). For example, from the parents’ perspective, their sons and daughters represent the ‘next generation’ and are two generations removed from their grandparents, thus forming a ‘biological chain’ (Spitzer 1973, p. 1354). This understanding is connected to kinship terminology (Pilcher 1994, p. 483) and relates to the broader realm of kinship relations (Kertzer 1983). Consequently, it is shaped by socio-cultural orders of kinship that can change and be violated, creating ambiguities and inconsistencies in the genealogical sequence of generations (see, e.g., Rovelli 2021, pp. 43–44). The notion of continuity involved in this genealogical understanding is also prevalent in discussions of value transmission, social mobility, and immigration (Kertzer 1983). Although it seems to focus on generations at the microsocial level, it actually refers to a broader societal context (Bengtson and Oyama 2010, p. 36). Such a genealogical concept of generations, based on reproductive lineage and one’s position in a reproductive cycle (e.g., as a parent, child, or grandchild), does not, per se, define a group. However, it points to certain similarities between and within the different generations. In the context of healthcare ethics, for example, the concept of “genetically related persons” (Brall et al. 2017, p. 30) who are affected by genetic information, and the accompanying idea of intergenerational relations, seems to evoke an understanding of genealogical generations that are connected by descent and inheritance. In the clinical context, issues of care responsibilities or proxy decision making within the family often rely on such an understanding of generations. In the field of research ethics, it becomes particularly prominent whenever research on genes and genetic data is addressed. Thus, in the ethical debate on germline interventions, the consequences of these technologies for future generations are considered especially from a genealogical perspective.

A *second*, *chronological conception* of generations refers to birth cohorts, or people born at roughly the same time (e.g., Rosow 1978). Following a primarily demographic approach, this conception stratifies people according to their year of birth; it “refers to the succession of people moving through the age strata, the younger replacing the older as all age together” (Kertzer 1983, p. 126). These approaches often claim objectivity and aim to establish the concept of generation as an important social category, comparable to gender, race, or class (Laufer and Bengtson 1974). An example is the “tobacco-free generation” initiative, where people born after a particular year would not be allowed to buy tobacco in order to successively eliminate the health risks of smoking in society (Kniess 2025). Still, interpretations vary. Some define birth cohorts narrowly in terms of a specific year. By contrast, a prominent chronological conception of Strauss and Howe distinguishes “cohort-group[s] whose length approximately matches that of a basic phase of life, or about twenty-two years” (Strauss and Howe 1991, p. 34). In this case, several adjacent birth cohorts form a distinct generation. In the context of healthcare ethics, this chronological conception is particularly prominent in debates on intergenerational justice and responsibilities for future generations. For example, discussions on what we owe to previous or subsequent generations, e.g., in terms of healthcare resources, usually seem to presuppose this notion, although the respective birth cohorts are frequently lacking concrete chronological specification (Munthe et al. 2021). In recent debates on the sustainable use of antibiotics, the focus is less on familial generations and more on future, unspecified birth cohorts that will be affected by antibiotic treatments today (Koesling and Bozzaro 2022).

The *third*, *socio-cultural conception* originates from the prominent description of generations as collectives shaped by shared historical experiences, socio-cultural influences, and social (self-)ascriptions (Mannheim 1928; Pilcher 1994) that result in similar attitudes, behaviors, and views within a generation (Costanza et al. 2012; 2021). Generations in this understanding appear as “the agents or ‘carriers’ of major cultural changes” (Berger 1960, p. 10) so that some refer to this strand as the “social forces approach to generations” (Laufer and Bengtson 1974, p. 183). It transcends cohort-based analyses, also encompassing the influence of the shared contemporary living environment on the members of a generation, and therefore takes a more comprehensive approach to localizing persons in their socio-historical context (Rosow 1978; Pilcher 1994). Labels such as ‘Generation Z’ or ‘Baby-boomers’ and the respective (self-)ascriptions refer to such a socio-cultural conception of generations and are, for example, applied and investigated in the Shell Youth Study (Albert et al. 2019; for a critical perspective, cf. Schröder 2018). This approach differs considerably from purely chronological models, as it is also possible that some birth cohorts cannot be assigned to any specific generation and that some generations may comprise more birth cohorts than others (Costanza et al. 2023). In healthcare ethics, for example, in the public debates during the COVID-19 pandemic, some referred to these approaches to explain the behavior of different age groups in the pandemic, e.g., when the experience of the post-war generation was used to explain their reluctant acceptance of help (cf., e.g., Ellerich-Groppe and Schweda 2025).^1^

These three conceptualizations of ‘generation’ are also rooted in and prevalent across different scientific contexts. Familial generations play a crucial role in pedagogy, youth and family research, but also bioethical debates on reproductive medicine or genetic research. In philosophical discussions about historical responsibility and intergenerational justice, chronological definitions of ‘past generations’ as those not living anymore or ‘future generations’ as those not yet existing are used (Golding 1972; Gardiner 2002; Thompson 2002; Gosseries 2008; Tremmel 2012; Meyer and Pölzler 2022). Socio-cultural conceptions of generations are frequently employed in sociological and cultural studies to examine character traits, attributes, and differences between generations (e.g., Costanza et al. 2012). However, it can also be advantageous to consider generational overlaps (Birnbacher 1988, pp. 23–27) or start from a description of a polity as an intergenerational continuum (Thompson 2009).

The generational viewpoint must be distinguished from the perspective of age groups. Age groups comprise individuals “who fall within a certain age range or are at a certain stage of life” (Daniels 1988, p. 12). By contrast, generations are usually characterized by their situatedness in temporal contexts beyond the individual life course, as becomes apparent in the above-mentioned theoretical strands. Age groups and generations can, in fact, overlap at a given point in time (Eisenstadt 2003), but they belong to diverging temporal orders and perspectives: while age group membership changes as an individual grows older, generational membership remains the same. Someone can belong to the group of the young at a certain point in time and transition to the group of the old at a later point. Still, their position in the genealogical order, their year of birth, or the fact that they experienced certain historical events and influences in their childhood and adolescence basically stay the same. In healthcare ethics debates, the confusion of both perspectives can have morally problematic consequences. Thus, the often interchangeable use of concepts of age groups and generations during the COVID-19 pandemic was frequently entwined with ageist stereotypes (Ellerich-Groppe et al. 2024).

This heterogeneous conceptual landscape explains why concepts of ‘generation’ have drawn extensive criticism regarding *theoretical definitions*, *methodological challenges*, and *actual uses* in public and scientific debates. First, comprehensive criticism refers to specific ways of *theorizing generations*. Especially the socio-cultural concept is criticized for assuming collective characteristics and promoting contingent labels that appear arbitrary and lack empirical foundation (e.g., Schröder 2018; Tremmel 2012, Chap. 2). Some even question whether such generations exist at all, and try to debunk the concept as a ‘myth’ (e.g., Schröder 2018). Beyond this theoretical uncertainty, *methodological challenges* are addressed. This includes difficulties in investigating a subject that lacks a concise definition, the comparability of (especially socio-cultural) generations, and the concept’s unclear added value vis-à-vis age groups and birth cohorts. Thus, several authors point to the methodological difficulties of disentangling the effects of ageing, cohort membership, and the historical period, the so-called age-period-cohort problem (Kertzer 1983; cf. also Riley 1973; Laufer and Bengtson 1974; Smith 2020). A third strand of criticism concerns the *use of the concept* in public and scientific debates, especially criticizing the reproduction of generational stereotypes (Costanza et al. 2012; Schröder 2018). Furthermore, there is criticism that the focus on generations and their differences obscures more fundamental issues, such as socioeconomic inequalities (Butterwegge 2009).

Especially with regard to healthcare ethics debates, there is also the concern that intergenerational approaches could distract from or even undermine prevailing individualist perspectives and frameworks. Individualism has a long tradition in medicine and healthcare, partly rooted in ethical responses to dehumanizing collectivist ideologies, policies, and practices, such as the historical atrocities of Nazi medicine and eugenics. Individualistic perspectives centered on personal autonomy, individual liberty, and human rights provide a powerful normative bulwark against such totalitarian tendencies. However, as illustrated by the examples of the sustainable use of antibiotics, data intensive medical research, and public health measures in a pandemic, focusing solely on individual rights, autonomy, and responsibility poses limitations. Here, intergenerational approaches can offer a complementary perspective to address specific challenges, such as healthcare claims of future generations. Indeed, the concept of generations has gained popularity in ethical debates not least due to its potential for articulating moral aspects that are easily neglected in strictly individualistic perspectives. *First*, the concept allows considering generational collectives as moral subjects or objects, i.e. their status as moral agents or addressees of moral actions. The definition and discussion of such collectives require critical ethical analysis, e.g., regarding underlying moral assumptions or problematic stereotypes and ageist discrimination. This also applies to their demarcation in time, that is, at what point in history a new generation begins or ends. Hence, the definition of the collective of generations already has normative significance, as generational definitions have implications for individuals, institutions, and society (Costanza et al. 2020; Costanza and Finkelstein 2015). *Secondly*, the concept opens the space to discuss the moral relations between different generations and to investigate the underlying narratives used to justify these relations, but also how moral claims between collectives are framed from a diachronous perspective. These ethical considerations include relations between coexisting generations, the effects of past generations’ behavior on the present and the future, and the moral significance of future generations for the decisions and actions of current ones. *Finally*, since generations as a cross-sectional category always comprise a certain degree of internal diversity, conflicts and moral relations within generations must be examined. This raises the question of the connection between intra- and intergenerational relations (Ellerich-Groppe et al. 2024; cf. also Yeh 2020). Related ontological and moral philosophical questions concern the role and contribution of the individual as a moral subject in the context of generations, and vice versa, the limits of generational perspectives vis-à-vis individual moral agency and responsibility (see also “Generations as collectives situated in time: towards a theoretically enriched understanding” and “Paradigms of intergenerationality: framing moral relations between generations” sections).

## Generations as collectives situated in time: towards a theoretically enriched understanding

Regardless of their differences, the three conceptualizations of generations introduced above share several commonalities: Generations usually refer to more than one person; talking about generations goes beyond a merely individualistic perspective and points towards a collective dimension (Sturn 2020). Furthermore, discussing generations involves considerations regarding temporal horizons, since the respective groups are essentially defined by their situatedness in time and their temporal interrelations. However, the dimensions of collectivity and temporality are interpreted in different ways in all three approaches, emphasizing different aspects regarding the composition, character, and resulting moral properties and relations of generations. Therefore, they ultimately also have different ethical implications as they envision different kinds of generational collectives regarding age, social, and political context, and devise different kinds of relations between these generations.

To exploit the potential of intergenerational perspectives in healthcare ethics, we need an explication, systematization, and reflection of the underlying ideas of generations. Without such a clarification, recourse to intergenerational perspectives loses its explanatory potential and runs the risk of giving way to crypto-normative or ideological claims. We aim to initiate this clarification from an ethical perspective. Since collectivity and temporality have proven to be crucial, we use these dimensions as a starting point for developing a theoretically enriched understanding and systematization of the concept of generation and of intergenerational relations. We distinguish an individualist, an aggregationist, and an entity view of collectivity, as well as a formal, linear, a subjective, existential-narrative, and a socio-cultural dimension of time.

### Collectivity: reflecting generations as collectives

When analyzing generational relationships, it is typically assumed that members of a given generation share a connection, whether explicitly or implicitly. Since a generation is usually not constituted by a single individual, the term ‘generation’ inherently refers to some form of social group or, generally speaking, to a collective.

Our consideration of ‘collectivity’ is not intended to replace but rather to complement perspectives focused on the individual. However, there are considerable debates regarding the meaning of ‘collective’, and a corresponding disagreement about the nature of collectives. Some scholars even question their very existence. Consequently, in applied ethics, the term ‘collective’ often remains vague, encompassing a wide range of social entities, such as families, research teams, political parties, professional organizations, nations, and, finally, entire generations.

For this reason, a closer examination of the nature of collectives is essential. Social philosophy and sociology have discussed this question extensively (e.g., Durkheim (1982 [1895], esp. pp. 31–59), Gilbert (1992), and Searle (2010, 90ff). We distinguish three main views of collectives which can help inform our analysis of ‘generation’: (a) *collectives as imaginaries*; (b) *collectives as aggregations of individuals;* and (c) *collectives as entities of their own kind* (for the latter two, see also French (2020)).

#### Collectives as imaginaries

A skeptical stance on the concept of collectives emerges from the individualistic conviction “that collectives do not exist in any philosophically interesting sense; that, even if collectives exist, they are incapable of moral agency” (Bunting 2009). This position is frequently shared by authors in the philosophy of economy who are committed to a (neo-)liberal framework. Some authors, such as Friedrich August von Hayek (1948) and those in line with this tradition, even advocate for a perspective that denies the existence of collective entities entirely.

The abovementioned forms of individualism regard concepts and ideas such as ‘society,’ ‘state,’ and ‘future generations’ as secondary or even irrelevant from an ontological as well as a moral standpoint. They either espouse the reductionist view that all actions and interests of these abstract entities can ultimately be traced back to individual actions and interests, or consider the idea of collective action and collective interests as such to be erroneous and misleading (for an overview, see Heath 2024). Consequently, these individualistic approaches operate without substantial notions of collectives, and collective dimensions are deemed to hold no normative significance. From their perspective, only individual rights, interests, or responsibilities are ultimately relevant.

Traces of this perspective can also be found in ethical discussions on generations, where it is often assumed that, also in intergenerational relations, individuals are the only relevant moral actors (Herstein 2009, p. 1180; Meyer 2018, p. 43). Moreover, viewing generations as superfluous imaginaries is echoed in criticism of generations as ‘myths’ (e.g., Schröder 2018), specifically targeting interpretations of socio-cultural generations as collectives with their own identity and shared generational attitudes and behaviors. However, this criticism of specific notions of generational collectives does not necessarily imply an individualist stance on all collectives.

#### Collectives as aggregations of individuals

The second position understands collectives as social aggregations of individuals in which intentions, will, and identity ultimately remain at the individual level. Understanding a collective as an aggregation of individuals does not deny its relevance *as a collective* and the group of people it encompasses. For example, it acknowledges the importance of cooperation between individuals (Isaacs 2011, pp. 26–27). Collectives are seen as a result of a socializing process, as indicated famously by the German term *Vergesellschaftung* (Lichtblau 2000, pp. 424 + 440).

There is an ongoing debate on whether and to what extent aggregative collectives can form collective intentions, bear collective responsibilities, and develop something like a collective agency, thus attaining the status of moral subjects. This is because as such an aggregative collective, an aggregation of individuals can develop majority views, even though there is no consensus on whether and in what way it might create a kind of ‘spirit’ of its own, based on shared considerations or plans (Pettit and Schweikard 2009, p. 578).

Although their status as moral subjects remains contested, aggregative collectives can function as moral objects, i.e., as a social category or a group of people who share normatively relevant characteristics and should therefore be taken into account in moral considerations. This is exemplified in contexts where chronological generations serve as normatively relevant reference points in contemporary ethical debates (Meyer and Pölzler 2022). An example is the discussion about the investment and innovation deficits of past and current generations regarding bacterial treatment options for future generations or even their use of antibiotics.

Especially aggregative views that group individuals based on socially effective attributions such as gender, disability, race, or ethnicity, can have powerful normative implications related to aspects of stigma, vulnerability, or discrimination (ten Have 2011; Hunter and Leverdige 2011). This is particularly relevant in health research and healthcare. For example, members of certain groups do not voluntarily share a collective identity per se, e.g., as patients with a particular disease. Still, they can affirmatively accept (or reject) such a grouping in the context of collective political activism (see also below).^2^

In practice, many quantitative arguments regarding majority opinions or future consequences for particular social groups also implicitly refer to such aggregative views. However, in a political or social movement, aggregations of individuals can transform into another kind of collective, for example, by forming we-intentions and developing rules that are constitutive of social institutions (Searle 2010, 42ff; for patient organizations, see: Beier et al. 2016). This might also hold true for generational collectives that develop a form of collective generational self-awareness. In this case, they become what can be called collectives as entities of their own kind.

#### Collectives as entities of their own kind

Viewing collectives as independent entities implies that they are ‘more’ and have another quality than the grouped individuals themselves. In other words, they are more than the sum of their parts and, hence, represent holistic-emergent phenomena (French 1982, 2020). From an ontological perspective, such collectives possess a distinct ‘mind of their own’ and a capacity for collective action based on communication and joint, collective decision-making (Pettit 2003).

Notable examples for this view can be found in the Hegelian and Marxian traditions, which postulate the existence of diverse and often stable collectives, such as social classes. Marx, for instance, describes the development of the proletariat from an aggregative collective into a collective entity in the course of the class struggle: “this mass is already a class, as opposed to capital, but not yet for itself. In the struggle, of which we have only noted some phases, this mass unites, it is constituted as a class for itself” (Marx 1913, p. 189).

The socio-political process of becoming a collective entity of its own kind has its own critical conditions—referring here to matters of collective identity, intention, and action planning. The dynamics of these conditions can be observed throughout the history of social movements, which often exhibit a collective oppositional orientation towards the established societal and political order to initiate social change (cf., e.g., Honneth 2005, pp. 162–164; 2003, p. 111).

While these fundamental ontological questions of the constitution of such collectives are undoubtedly important, it is evident that they do not automatically clarify all ethically relevant issues, such as whether these collectives are formed voluntarily or involuntarily (Schicktanz and Buhr 2021). The voluntary formation of a collective is based on communication and participation, the deliberate development of common interests, intentions, and actions (Bratman 1992). Examples include political groups (e.g., activists fighting for or against certain developments in the healthcare system), professional organizations in the healthcare sector, or ethics committees.

In medicine, for example, involuntary collectives become manifest in family relationships. These can be significant not only in a biological sense due to genetic predispositions to illness but can also result in morally relevant decision-making processes in parent-child relationships. Additionally, social commitments to a group are tied to a “norm of stability” (Bratman 1992), and its identity can persist only if its members do not abandon their common intentions. Such stable groups can act, claim interests, or be held responsible in a manner different from unstable or transient groups.

From a normative perspective, however, the question of collective agency remains the most pertinent one. It is, therefore, necessary to consider whether it is possible to speak meaningfully of collective autonomy, collective responsibility, or collective solidarity. In applied ethics, this calls for an assessment of whether and to what extent the collective in question can act beyond the limits of mere individual action. Additionally, it is essential to ascertain whether the collective has established rules and practices of cooperation, of forming collective agency.

With regard to intergenerationality, such an entity perspective on collectives, as well as the accompanying moral philosophical questions, become manifest in the (re)negotiation of moral relations between generations. Thus, in the ascription and sometimes self-attribution of socio-cultural identities in public debates, generational collectives appear as more than mere aggregations of individuals, acting instead as entities of their own kind. For example, addressing the ‘Boomers’ as contributing to climate change which ‘Generation Z’ or ‘Generation Alpha’ have to face reflects at least some idea of generational collectives as entities interacting across time.

### Temporality: aspects of a differentiated understanding of time

Despite their crucial importance, the complex meanings of time and the structures of temporality have received relatively little systematic attention in healthcare ethics (Bernegger et al. 2012; Wiesemann and Schweda 2023; Schweda and Wiesemann 2016). The introduction of intergenerational perspectives into healthcare ethics requires a more explicit and nuanced understanding of time and temporality.

All ideas of generations assume some form of temporal simultaneity. Members of a generation usually live at the same time in a specific historical context and are approximately of the same age (Jansen 1974). This brings into focus broader temporal frames, since the relevance of the past for the present and the present as past in the future almost inevitably becomes concrete in the debate about generations. Some even view generations as a way of structuring organizations and societies in a temporal dimension (Struck 2004). For example, already in the 1980s, Matthes proposed to talk about generational conditions, “in which the temporal structure of social events is ‘polyphonically’ organized” (Matthes 1985, p. 369; own translation). Generations thus offer a framework for addressing the “multifaceted nature of time” (Pilcher 1994, p. 488), for example, by considering a succession of familial generations, the ‘before and after’ of specific birth cohorts, or the formative force of historic events regarding socio-cultural generations. In contrast to the widespread ‘ethical presentism’ in healthcare ethics debates, where the present often appears as the ‘standard time zone’ of ethical reasoning (Schweda and Bozzaro 2014), time has a more complex and multidimensional structure in intergenerational contexts. On the one hand, the past plays a crucial role in understanding present moral claims and conflicts. For example, in the healthcare system, contemporaries have inherited historical responsibilities due to reprehensible research or traditions of sexism and racism that influence their current understanding of needs, distrust, or healthcare inequalities (Schicktanz and Stoff 2021). On the other hand, assumptions and normative expectations about the future influence how present decisions and actions are perceived and evaluated. For example, decisions about prioritizing investments in biomedical research over other fields depend on the perceived future value of health and health promotion. We believe that a differentiation between three basic views of temporality can be helpful in discussions about intergenerational relationships: (a) *formal*,* linear time*; (b) *subjective*,* existential*-*narrative time*; and (c) *socio-cultural time*.

#### Formal, linear time

One of the first definitions of time in Western philosophy can be found in the fourth book of Aristotle’s *Physics* (Aristotle 1983). Here, time is defined as the number of movements in relation to a ‘before’ and an ‘after.’ In this sense, time is a countable quantity. For the distinction between a before and an after to exist, there must be a dividing point between them; this is the moment.

This formal concept is related to a linear understanding of time as a never-ending succession of time points. This sequence is structured by the definition of time units, such as minutes, days, weeks, and years, and is measured by clocks, calendars, and similar devices. It counts oscillations of similar length and is an artifact in the same way that clocks are artifacts that measure them (Adam 2006, p. 101). This formal concept of time provides an objective frame of reference for organizing human activity at both individual and societal levels in a decontextualized and merely quantified way.

The formal, linear concept of time is related to the first and second interpretations of generations mentioned earlier. The genealogical sequence of generations, based on reproductive cycles, is usually expressed as a linear and potentially infinite sequence. In a similar way, the chronological concept of generations relies on a formal, linear concept of time where generations are defined and distinguished by birth years. In this understanding, generations are located on the temporal continuum based on their year of birth and are formally related to each other in terms of ‘before’ and ‘after.’

With regard to the field of healthcare, this concept of time is omnipresent in clinical settings: The year of birth constitutes a standard element of a patient’s record and the question of chronological age plays an important role, for example, in the definition of ‘age-typical’ or ‘age-atypical’ functioning, which is essential to prominent disease concepts (cf. Boorse 1975). Furthermore, chronological age plays a role in clinical practice and decision-making: Minors usually cannot give legally valid informed consent, and special safeguards accompany their participation in clinical trials. In public health, chronological age serves as an explicit or implicit criterion for the rationing of healthcare resources in many countries. These examples show that a linear, formal concept of time can have significant normative consequences.

This concept of time is also central to some of the ethical challenges of intergenerational relations mentioned earlier. For example, the demand for sustainable use of antibiotics, or of healthcare resources in general, can only be justified if we assume that there is a future linked to the present in a linear time continuum so that actions taken today will have consequences for future patients. A controversial issue in this context is whether and to what extent time horizons are relevant from a normative perspective: Should present people only be held responsible for consequences that may arise from their actions in the next 50 to 100 years (the grandchildren argument), or is it also appropriate to consider longer time horizons? Regardless of how one answers this question, a discourse on sustainability only makes sense if one assumes, in a linear time perspective, that there will or should be future generations in the sense of descendants or future birth cohorts in general (Becker 2012).

#### Subjective, existential-narrative time

Implicit in the Aristotelian definition of a formal, linear time is that the perception of movement from a ‘before’ to an ‘after’ presupposes a unified observer’s perspective capable of organizing events in their before-and-after relationship. In his *Confessiones*, Augustine attributed this synthesizing function to the human soul (Augustine 2006). He also defined time as the number of movements, but he meant the movements of events that occur in the human mind through memory, attention, and expectation.

At least in the Western context, human beings are conceived not only as living beings who can consciously relate to points in time but also as beings characterized by the need to relate to their own finite and transient lives. In *Time and Free Will* (Bergson 1913), Henri Bergson defended a lived time as ‘duration’ against chronological, linear time. Its intensity, for instance, emerges in the unfolding experience of an illness and cannot just be measured by clocks and calendars. Paul Ricœur reflected on individuals’ relationship to their own lifetime under the heading of narrativity. According to him, a narrative develops as a continuum that presupposes a beginning, a middle, and an end (Ricœur 1984). It is never a simple chronicle, i.e. a punctual and straightforward itemization of a series of events. Despite the diversity and heterogeneity of individual storylines, every narrative presupposes unity in the sense of a coherent plot. The plot holds the individual events together, thus distinguishing the narrative’s chronology from the actual events’ chronology. While the latter is a sequence of individual moments, the narrative’s chronology is organized according to the dramaturgical discretion of the narrator. It can thus be actively shaped and is meaningful.

This subjective, existential time is connected to the first, genealogical understanding of ‘generation’ in that people generally perceive and narrate their position within a (linear) timeline precisely through their role in certain (family-)narratives. One’s identity is shaped by a history of origin from which one cannot free oneself completely. This aspect also plays a vital role in medicine: The medical history (anamnesis), for example, comprises the reproduction of a family history (previous diseases, genetic risk profiles, etc.). In psychoanalysis, too, the recollection of one’s origins is crucial. However, this existential time perspective has long been neglected in medicine and healthcare contexts that traditionally focus on objective time measures. For example, the linear time of reproductive medicine, with its treatment cycles and age limits, differs from the temporal experience of couples living in specific social settings that confront them with somewhat different time ideals. The biologically ideal time to conceive a child is not the same as the socially ideal time (Rimon-Zarfaty and Schweda 2018).

It is important to acknowledge that especially experiences of illness or aging can profoundly affect the subjective perception of time. People with a chronic disease, for example, are often forced to change their usual daily routine because of the symptoms of their illness. They may no longer be able to maintain their normal rhythm of life because the intensity of the disease is constantly fluctuating and they have no control over it. They must continuously adapt to their illness’s “time rhythms” (Charmaz 1991). The changes in the subjective perception of time in some mental illnesses are particularly impressive: Severely depressed patients experience time as an extended present that does not seem to pass, with a blocked future horizon (Fuchs 2013). This aspect is crucial for helping chronically ill people live a good life with their disease. Another example of the importance of individual time horizons is end-of-life decision making. Patients often want to continue therapy until a significant event arrives, such as their granddaughter’s birthday. Taking such time horizons into account is essential for good clinical care.

This connection between life, time, and narrative becomes particularly interesting from an ethical point of view when we consider that individuals are both protagonists of their stories, as they live their present time, and their editors, as they decide how to live this time. Human actions and lives are not simply given objects or facts. They unfold over time and thus have a particular temporal extension and structure that is relevant to their ethical evaluation. The specific form of processuality of human action and life additionally requires a closer examination of the moral significance of related temporal concepts such as directionality, irreversibility, and finitude. Individual lives and historical developments are ultimately unidirectional and cannot be reversed or undone. This is essential for our understanding of fundamental moral concepts such as guilt, responsibility, or compensation, which may be pertinent in the lived reality of decision-making in intergenerational family healthcare.

From an intergenerational perspective, the subjective, narrative dimension of time is significant in that it allows to connect individuals and groups in a meaningful way through memory, attention, and anticipation. It transcends the mere chronology of a series of points on a linear timeline and creates morally relevant connections, entanglements, and networks between them. In the contexts of genetics, psychoanalysis, or resource allocation, for example, what one inherits from one’s ancestors and what one passes on to one’s descendants, both in a positive and in a negative sense, is of crucial moral importance. The localization of one’s own position in a generational sequence is, therefore, at the heart of the attribution of intergenerational claims and responsibilities.

#### Socio-cultural time

In the development of an individual life narrative, individuals are dependent on physical and biological processes, such as the onset of the reproductive phase, growth, and aging processes, which determine the framework within which they can shape their own lives in accordance with personal ideals and values. At the same time, social and cultural time concepts and norms also influence how individuals make life choices. There are more or less rigid social ideals of what an ideal life course should look like. These define time windows within which certain decisions or developmental steps should occur, such as education or reproduction, or when certain behaviors appear appropriate (Schweda 2017).

This time dimension is related to the third, socio-cultural concept of generation. Thus, Abrams observes a “mutual phasing of two different calendars” (Abrams 1982, p. 240) in the socio-cultural notion of generation, where the stage in the life cycle is set in relation to the socio-cultural time (cf. also Pilcher 1994, p. 488). After all, health-related behavior reflects not only an individual choice and idiosyncratic lifestyles but also social norms, ideals, and expectations. For example, while smoking was largely taken for granted by large parts of the so-called ‘Baby-boomer’ generation, the emphasis on a healthy work-life balance is just as much a matter of course for the so-called ‘Generation Z’. Generations in a socio-cultural sense are believed to have shared ideals of health and a healthy life. This is not surprising when one considers that the apprehension of health-related topics depends on the contemporary state of knowledge: While infectious diseases were seen as a significant challenge 100 years ago, lifestyle-related chronic diseases, such as cardiovascular diseases, dominate current public health debates.

From an intergenerational health perspective, it is necessary to reflect on the choices made by one generation and how they might impact those that follow. Today’s generations in Western countries have inherited great prosperity, but they are also increasingly concerned about the present and future climate and environmental crises. Decisions about pension systems and long-term care regimes established over 50 years ago in the context of demographic growth could hardly be made in the same way now, given current ageing populations. Future generations will have to live with the consequences of decisions made today.

Here, narratives also play a central role, providing a common form to describe and explain the entwinement of past, present, and future perspectives, as well as the unfolding of processes over time. They typically involve a specific, morally relevant interpretation of actions, events, and their interconnections (Forst 2013). This is true at both the individual and societal levels, where shared socio-cultural narratives exist. Depending on how we tell the story of a set of past events, such as past generations’ decisions regarding the use of antibiotics or participation in medical research, different moral claims and liabilities will arise for the present and the future. Frequently, cultural traditions and media discourses provide overarching narratives, such as those of medical progress or cultural decline, that convey particular moral perspectives. They, therefore, need critical ethical reflection (Ellerich-Groppe and Schweda 2025).

## Paradigms of intergenerationality: framing moral relations between generations

An adequate consideration of generations in healthcare ethics requires theoretically robust and normatively reflective perspectives on collectivity and temporality. While various notions of ‘generation’ exist, they all have in common that they refer to certain kinds of collectives characterized by their specific situatedness in time. Hence, different notions of generations are based on different understandings of collectivity and temporality.

These understandings of collectivity and temporality hold ethical significance as they frame how moral relations between generations are conceived and discussed in healthcare ethics. In the following, we highlight three typical approaches to intergenerational relations to illustrate the scope of conceptualizations of generations, the implied understandings of collectivity and temporality, and the corresponding ethical questions and problems: (1) a focus on temporally stratified individuals that raises questions regarding the normative relevance of past or future aggregative collectives and their temporal proximity; (2) the idea of collectives in allegiance or conflict, which touches upon questions of intra- and intergenerational cohesion and connectivity; (3) the notion of transgenerational communities that highlights the relevance of the continuity and interdependence of generations for the flourishing of an overarching polity.

These considerations outline paradigmatic perspectives on moral relations between generations. They do not claim to be exhaustive and do not preclude other interpretations and constellations. We pick them to illustrate three key points: First, debates in intergenerational healthcare ethics need to articulate the underlying concepts of generations. Secondly, for this purpose, the dimensions of collectivity and temporality prove to be essential. Thirdly, intergenerational perspectives in healthcare ethics are productive as they allow to address important points of analysis for the field. In order to elucidate each of the following paradigms of intergenerationality, we will refer to the introductory examples from the fields of clinical practice, biomedical research, and public health.

### Relations between temporally stratified individuals

A first understanding of moral relations between generations is based on the notion of temporally stratified individuals. It starts from an individualist or aggregationist perspective on the generational collective, e.g., in terms of birth cohorts. The temporal focus lies on intertemporal relations between individuals based on a chronological understanding of time.

The debates about an impending post-antibiotic era can serve as an example. The excessive use of antibiotics leads to the spread of antimicrobial resistance, jeopardizing the long-term preservation of effective antibiotic treatments. As a result, the possibility of treating bacterial diseases in future generations of patients will be limited, resulting in fewer medical treatment options than currently available (Koesling and Bozzaro 2022; Bozzaro and Koesling 2023).

Upon closer inspection, this case highlights fundamental aspects of ethical debates regarding the consequences of today’s decisions and actions for future generations. Such debates often evoke a chronological *understanding of generations* as birth cohorts whose demands and needs must be considered and balanced. As such, they raise several theoretical questions that require further consideration and clarification.

Regarding *collectivity*, the view of generations as birth cohorts involves an aggregationist view of the generational collective. The individuals living today are summarized as one cohort relying on the use of effective antibiotics. This can obscure existing disparities, e.g., between the Global North and the Global South, that may suggest different claims and responsibilities among present generations. By contrast, future generations are addressed as unspecified groups of not-yet-existing individuals possibly endangered by a lack of effective antibiotics. It appears much more challenging to anticipate their healthcare needs. Thus, the talk of future generations ultimately addresses an abstract class of potentially affected individuals without concrete characteristics.

Regarding *temporality*, the discussion invokes the idea of a formal, linear timeline as a never-ending succession of equally relevant points in time. When discussing the post-antibiotic era, we are thus comparing the present point on this timeline with a largely unspecified future period without a closer look at existential and socio-cultural aspects. This leaves open the normative significance of the implied generational structure and segmentation of the future, for example, whether our moral responsibilities towards individuals in the near future differ in kind or degree from those towards individuals in the more distant future.

The perspective of temporally stratified individuals ultimately frames all relevant responsibilities as individual responsibilities. Hence, it does not rely on controversial ideas of collective agency and collective responsibility; the individuals remain the relevant moral agents (Herstein 2009, p. 1180; Meyer 2018, p. 43). At the same time, this stance must make assumptions about the needs and problems of not-yet-existing future individuals and thus raises questions about the empirical basis and theoretical plausibility of such forecasts. The example thus highlights the challenges to determine the moral relations between non-overlapping generations. In moral philosophical contexts, this also raises complicated issues regarding the moral evaluation of our present actions for future persons, such as the non-identity problem (Parfit 1984), anticipatory gaps (Rueda et al. 2025), and questions of temporal discounting (Portney and Weyant 2013). Therefore, the normative contribution of the concept of generation in such assessments needs further clarification.

### Collectives in allegiance or conflict

A second paradigm views generations as co-existing or overlapping collectives in allegiance or conflict. Such a perspective evokes the common notion of a battle of generations or of open invoices between generations (e.g., Kohli and Kühnemund 2005; Müller-Salo 2022). For example, Jansen (1974) paints a woodcut picture of the relationship between generations when she assumes a hegemony between the generations existing at the same time that are therefore in cohesion, tension, or conflict. Following her, the socio-historical reality is a result of „the dialectic and tensional relations obtaining between the coexisting generations of the time” (Jansen 1974, p. 96).

This perspective usually involves an *understanding of generations* as collective entities in their own right. They are not just aggregative cohorts of individuals born in the same year, but genealogically connected groups (e.g., in the pertinent works of Bengtson and colleagues on intergenerational solidarity (Bengtson and Roberts 1991; Silverstein and Bengtson 1997)), or socio-cultural generations based on shared socio-historical experiences that result in common characteristics. Regarding the temporal dimension, certain past or expected future events can play into the ethical considerations, as they are constitutive for the respective generations and their moral entanglements.

The debate on the COVID-19 pandemic can serve to exemplify this framing. During the pandemic, the idea of intergenerational solidarity functioned as a prominent yet controversial normative reference point for the justification of public health measures (Ellerich-Groppe et al. 2024). For example, social distancing, isolation, and lockdowns were often justified on the grounds of intergenerational solidarity with the vulnerable group of older people. Others asked older generations to set aside their own needs and isolate themselves to allow the younger population to continue living their lives (Ellerich-Groppe et al. 2024; Rudolph and Zacher 2020).

In this case, the underlying notion of *collectivity* appears ambivalent: often, age groups with different needs and vulnerabilities are identified or conflated with generations, thereby fueling age stereotypes and ageism (e.g., Ayalon et al. 2021; Rudolph and Zacher 2020). At the same time, we also find allusions to genealogical or socio-cultural understandings of generations as collective entities. For example, the old were sometimes identified with the grandparents (#stayhomeforgrandma) or the ‘Boomer’ generation, and the question of their protection during the pandemic was thus connected to intrafamilial responsibilities or previous debates about intergenerational justice in the context of climate change (Ellerich-Groppe et al. 2024; Rudolph and Zacher 2020). Conversely, ‘generation’ also served as a category of self-appropriation, e.g., when individuals identified as ‘Boomers’ or members of the ‘Generation Corona’ and derived claims or responsibilities from this membership (cf., e.g., Ellerich-Groppe et al. 2024; for an example from the German media debate, see also Behbehani 2021).

This is where the dimension of *temporality* comes into view: On the one hand, the perception of the pandemic as an emergency and an urgent challenge promoted a focus on the immediate present. On the other, the accompanying appeals to intergenerational solidarity frequently rested on the narrative evocation of shared past experiences that had shaped collective generational identities. Furthermore, narratives about the previous achievements, failures, and entanglements of these generational collectives also provided the basis for intergenerational claims, responsibilities, or liabilities (Ellerich-Groppe and Schweda 2025). Eventually, many of these narratives suggested that the pandemic would also have consequences for the future—and, in this way, for the collective identity of future generations that would have to deal with the economic and social fallout of contemporary public health decisions and actions.

Such a perspective allows to understand moral relations over time beyond individual perspectives and, therefore, to consider broader socio-cultural developments. It adds ‘generations’ as another analytical social category (such as gender, class, and race) and enriches our understanding of society, enabling a nuanced view of intra- and intergenerational conflicts and moral responsibilities. At the same time, the discursive construction of generations as entities raises questions. Interpreting generations as actors in moral conflicts presumes a notion of collective identity, collective responsibility, and collective agency. Furthermore, the idea of a battle of generations runs the risk of establishing dichotomous distinctions between allegedly homogeneous generational collectives based on social practices of (self-)ascription and othering. As age groups and socio-cultural generations can be considered at least involuntary, if not coerced collectives, this has far-ranging consequences for their individual members. The underlying entity view of generations constructs moral agents that are in relation to one another. Finally, despite promoting a broader time frame, this approach primarily focuses on past and present generations while offering a somewhat limited perspective on the future.

### Transgenerational communities

A third paradigm discusses relations between generations in terms of a transgenerational community. Such a model assumes that the interdependence of different generations is relevant for the flourishing of a polity over time, similar to the idea of a diachronic “intergenerational continuum” of overlapping, connected generations (Thompson 2014). Due to this focus on a specific continuity, such models primarily involve genealogical *understandings of generations* that can contribute to the transgenerational community. Here, different time frames overlap, particularly subjective and socio-cultural time, since such a community presupposes a specific shared context. However, the perspective goes beyond (‘trans’) the notion of a present community of overlapping generations as it also involves past and future generations.

The debate about data-intensive biomedical research can help clarify the respective moral implications. Such research is based on the collection, integration, and evaluation of heterogeneous data sets: genetic data, so-called omics data, data from clinical care, environmental data, and lifestyle data. The willingness of patients and citizens to provide health and treatment data for research purposes also requires long-term involvement. Research may not benefit current patients, but it may serve future generations by preparing the ground for new therapies. This raises intergenerational questions.

In this context, traditional ideas of a chain of generations or the prevention of harm to future generations are prevalent. With regard to *collectivity*, the idea of genealogical generations often involves a notion of a solid, quasi-natural collective entity shaped by the linear time of genealogical ‘bloodlines’ or the cyclical temporality of reproductive rhythms. Regarding research ethical questions, this genealogical perspective becomes particularly clear when considering research on genetic data, where every intervention or research finding has immediate implications for subsequent generations (cf. also Brall et al. 2017; Raz and Schicktanz 2016). For example, while some argue for more research on genetic data and genome editing to be able to effectively treat diseases in the future, others reject the use of these technologies due to the unclear consequences of such interventions for the human genome (Savulescu et al. 2015; Schleidgen et al. 2020; Lorenzo et al. 2022). Yet, the respective responsibilities of every generation need clarification. While some may emphasize specific unidirectional responsibilities, others might instead stress mutual responsibilities between generations within the transgenerational community, such as notions of intergenerational contracts. Both perspectives suggest a specific interdependency between different generations.

Within this transgenerational model, *temporality* extends beyond the present point in time, encompassing both the heritage of past generations and the concerns of future generations. The question is how to deal with medical data within a larger, collective and trans-temporal horizon without neglecting other ethical principles, such as individual autonomy, care, and justice. The idea of a transgenerational community leaves room for different time horizons between the subjective, existential-narrative and socio-cultural time frames. For example, I may be interested in the health of my own descendants (reproduction) while at the same time wanting to avoid the larger societal consequences of these interventions for future generations. More fundamentally, we need to ask for which temporal scope an individual can be held responsible in this transgenerational community, and what time frames transcend individual responsibility (Portney and Weyant 2013). At the intersection of temporality and collectivity lies the further question of how to balance one’s responsibility, and whether the responsibility for existing generations or future generations weighs more.

The concept of a transgenerational community contradicts the view of non-overlapping generations, which is predominant in healthcare ethics and political philosophy and is exemplified, for instance, in so-called ‘longtermism’ (Crary 2023). The notion of non-overlapping generations idealizes the relations between generations as ‘purely’ intergenerational (Gardiner 2002), assuming that generations are temporally separate collectives. The idea of a transgenerational community, by contrast, allows a specific consideration of the concept of generation in ethical assessments and focuses on continuity over time. It can be understood as an approach to integrate an empirically substantial and normatively relevant idea of generations that nevertheless leaves space for a context-dependent determination of responsibilities. At the same time, this approach extends individualistic moral philosophical concepts such as ‘moral actor,’ ‘agency,’ and ‘responsibility’ to a collective level and thus needs further clarification (Rehmann-Sutter 2025). For example, it is controversial whether and under what conditions collectives, such as generations, can be suitable subjects of responsibility. After all, assuming responsibility presupposes certain features of personhood, such as intentionality and self-determination.

## Conclusion: what intergenerational perspectives can contribute to healthcare ethics

Current challenges in medicine and healthcare highlight moral relationships between generations. Yet, healthcare ethics lacks a systematic approach to the complex and ambiguous category of generations. Our considerations aimed to clarify this category and thus to identify ways in which intergenerational perspectives can make a valuable contribution to healthcare ethics.

We first distinguished three main theoretical strands of how generations can be conceptualized: Genealogical approaches focus on groups connected by relations of descent and kinship. Chronological approaches define generations according to birth cohorts. Socio-cultural approaches understand generations as collectives shaped by shared historical experiences and socio-cultural meanings. In all of these strands, the dimensions of collectivity and temporality proved to be decisive. A theoretically enriched understanding of these two dimensions allows the identification and characterization of paradigmatic perspectives on intergenerational relations in healthcare ethics (cf. Table 1). The ideas of temporally stratified individuals, of collectives in allegiance or conflict, or transgenerational communities not only refer to different generational concepts but also convey different understandings of collectivity and temporality.

**Table 1:** Overview of ideal-typical paradigms of intergenerational relations

Intergenerational relations	Temporally stratified individuals	Collectives in allegiance or conflict	Transgenerational communities
Understanding of generation	Chronological generations	Socio-cultural generations	Genealogical generations

Dominant dimension of collectivity	Aggregationist view	Entity view	Entity view

Dominant dimension of temporality	Formal, linear time	Socio-cultural time	Formal, linear time
			Subjective, existential-narrative time

The discussion of these paradigms highlights the potential of intergenerational perspectives in healthcare ethics. Thus, they expand the scope of possible moral subjects and objects beyond the individual level. Especially in challenges such as the climate crisis that cannot be discussed adequately based on individual behavior alone, generations may offer a complementary viewpoint without blocking the discussion of the role of the individual. Furthermore, intergenerational perspectives also enable us to consider overarching time horizons in a systematic way. Just as they make it possible to assess the present use of antibiotics in relation to its prerequisites in the past and its consequences for the future, they re-contextualize research ethical questions in a temporal dimension, providing new starting points for a time-sensitive assessment that overcomes ethical presentism. Finally, by systematically combining the dimensions of collectivity and temporality, intergenerational perspectives offer an integrated approach. In this way, they can provide another “structural category with the same kind of analytic utility as a variable like social class” (Ryder 1965, p. 847) that can serve as a lens for healthcare ethical investigation.

At the same time, a fruitful discussion of intergenerational issues in healthcare ethics depends on several conditions: First, the analysis has shown that a systematization, explication, and ethical reflection on the respective underlying concept of generations are indispensable prerequisites for a meaningful and consistent application of intergenerational perspectives. In this regard, the three theoretical conceptualizations of generations identified above can provide a starting point. Secondly, our considerations underscore the need to rethink normative concepts and principles applied in intergenerational perspectives in terms of collectivity and temporality. Common bioethical concepts, such as the four principles of biomedical ethics, could benefit from a reconsideration through the (inter-)generational lens of collectivity and temporality. This allows to expand the view beyond the domain of professional ethics to include healthcare and public health ethics perspectives. Similarly, concepts like responsibility, solidarity, and sustainability that are increasingly used in intergenerational perspectives (cf., e.g., Munthe et al. 2021; Yeh 2020) and may show a stronger affinity to dimensions of collectivity and temporality might gain additional normative potential from a reflective understanding of these dimensions and of the overarching idea of generations. Thirdly, the role and relative contribution of intergenerational perspectives must be considered in the context of existing approaches in healthcare ethics. In particular, they are not to replace individualistic perspectives, but rather to productively expand and complement them, drawing attention to issues that are at best indirectly considered in individualistic frameworks.

This contribution addresses only a fraction of the questions and problems that arise from (inter-)generational perspectives in healthcare ethics. Thus, it is essential to consider more elaborate understandings of collectivity and temporality in *clinical ethics* debates and broaden the individualist and synchronous perspective of the prevailing principlist framework. For example, a comprehensive understanding of sustainability could help clarify the moral implications of current clinical practice for future generations in a more adequate manner, not only with regard to the antibiotic crisis, but also to issues of green healthcare (De Maack and Dupras 2025). Furthermore, current debates in *research ethics*, where the protection of individual rights is contrasted with future benefits for society (e.g., Savulescu et al. 2015; Mintz et al. 2019), can benefit from a closer analysis of the risks and chances of medical research for current and future generations. An analysis of the collective responsibilities of researchers also enables us to shed more light on issues of sustainability and future-oriented distributive justice related to research practices, research integrity, open science claims, and research infrastructure. In *public health ethics*, the moral issues arising from intergenerational relations during crises like the COVID-19 pandemic can be better addressed through a nuanced understanding of social group differentiation and normative framework conditions of rational-contractualist or more emotional moral bonds between generations. Here, it can be productive to take into account the narrative construction of (self-)ascribed generational identities based on shared experiences and moral entanglements among members of different age groups (Ellerich-Groppe and Schweda 2025).

As we have pointed out, the emerging debate about intergenerational healthcare ethics requires more systematic research, in-depth analysis, and further elaboration. It touches upon fundamental theoretical and empirical questions about the ontological or moral status of collectives, and the structure and normative relevance of temporal relations. These comprise the interdependencies between individuals and generations, and between intra- and intergenerational relations, the moral status, agency and responsibility of generational collectives, as well as the normative relevance of processes and perspectives that transcend the present moment and the temporal horizon of individual time.

## Footnotes

1 On the threshold between chronological and more substantive, socio-cultural approaches, Norman B. Ryder defines generations as neighboring birth cohorts that grow up in—and are therefore affected by—the same social, political, and structural conditions. The size of the own cohort and the relation to others are also seen as relevant factors for the life of the individual members. Following Ryder, this concept of cohorts allows to identify or rather reconstruct “composite cohort biographies” that can be compared and help to observe social change (Ryder 1965, p. 847). This definition allows Ryder to understand cohorts as a “structural category with the same kind of analytic utility as a variable like social class” (ibid.).

2 It can even result in categorizing others as ‘different from us,’ which is often pejorative. For instance, in intergenerational discourse, the ascription of chronological age can categorize individuals of an advanced age as belonging to one generation, while younger individuals are designated as the ‘younger generation X, Y, or Z,’ for example, based on their criticism of the habits or values of the former. This type of categorization can manifest in exclusionary practices such as ‘othering’ (Spivak 1985), which are seen as problematic attributions.
